# Modeling the air-soil exchange, secondary emissions and residues in soil of polychlorinated biphenyls in China

**DOI:** 10.1038/s41598-017-00351-0

**Published:** 2017-03-16

**Authors:** Song Cui, Qiang Fu, Yi-Fan Li, Jianmin Ma, Chongguo Tian, Liyan Liu, Leiming Zhang

**Affiliations:** 1 International Joint Research Center for Persistent Toxic Substances (IJRC-PTS), School of Water Conservancy and Civil Engineering, Northeast Agricultural University, Harbin, Heilongjiang 150030 China; 2 IJRC-PTS, , State Key Laboratory of Urban Water Resource and Environment, Harbin Institute of Technology, Harbin, Heilongjiang 150090 China; 3 IJRC-PTS-NA, Toronto, M2N 6X9 Canada; 4 Key Laboratory of Western China’s Environmental System, Ministry of Education, College of Earth and Environment Sciences, Lanzhou University, Lanzhou, Gansu 730000 China; 5 Yantai Institute of Coastal Zone Research, Chinese Academy of Sciences, Yantai, Shandong 264003 China; 6 Air Quality Research Division, Science and Technology Branch, Environment Canada, 4905 Dufferin Street, Toronto, Ontario M3H 5T4 Canada

## Abstract

The present study investigated the environmental distribution and fate of low molecular weight (LMW) polychlorinated biphenyls (PCBs) in China using the ChnGIPERM (Chinese Gridded Industrial Pollutants Emission and Residue Model), in which the air-soil exchange, spatial-temporal variations and the heterogeneity of secondary emission and residue in the non-source areas were studied. The model simulated the temporal and spatial variations of the PCB28 concentration in soils and air which agreed well with historical monitoring data across China. The long-range atmospheric transport (LRAT) and temperature was identified as the major factor affecting the distribution patterns of the secondary emissions and residues. Soil residue was considered as important environmental fate of PCB28. However, the intensity of an emissions source and the distance with non-source area strongly affected the spatial and temporal variations of PCB28 residues in soil. Several factors strongly impacted the distribution characteristics and air-soil exchange of PCB28, including emission patterns, atmospheric transport, soil organic carbon (SOC), soil vertical transfer, ambient temperature, and precipitation.

## Introduction

Persistent organic pollutants (POPs) are classically characterized by their environmental persistence, high toxicity, and potential to bioaccumulation in food chains. Their long-range atmospheric transport (LRAT) has resulted in their widespread global distribution^1^. Polychlorinated biphenyls (PCBs) are priority controlled as one of twelve legacy POPs targeted by the Stockholm Convention on POPs^2^. Historically, PCBs, which have no natural sources, are synthetic chlorinated organic chemicals that were mostly applied in the products such as transformers and capacitors due to their thermal stability, excellent dielectric properties, and resistance to oxidation^3^. PCBs have been banned for several decades but released continuously from old equipment and waste sites^4, 5^. Atmospheric transport is the major dispersion and transfer pathway for PCBs and has widely distributed these compounds all over the world from the sources to remote regions. Extensive studies have been conducted to elucidate the contamination of PCBs in different environmental media such as the atmosphere^6, 7^, soil^8–11^, water^12^, sediments^13–15^, and biota^16–18^.

Acting as a natural reservoir or sink and also a secondary emission source in the terrestrial environment because of the abundant organic matter (OM) content, soil plays an important role in the environmental distribution and cycling of PCBs. The soil burden is often used to interpret the historical emissions, atmospheric deposition, and fractionation effect of POPs^19–22^. Differing from pesticides which are used in agricultural crops, PCBs are used in many commercial products. After entering the environment, PCBs can be redistributed on a global scale by the effect termed “global distillation”^23^. In the process of redistribution, however, LRAT acts as a “carrier” that moves the chemicals from warmer regions to colder regions or from the places of source to remote areas, accompanied by dry and wet deposition, which are the most important pathways of the migration of PCBs into each environmental compartment^24, 25^. The chemicals undergo several cycles of deposition and re-emission before reaching relative steady environmental media, a process termed “grasshopping”^24^. The status of POPs in the soil and air is mainly controlled by air-soil exchange^26^. The compounds continuously exchange between the atmosphere and terrestrial environment through recycling over a long period of time before achieving steady state between the environmental media^27^.

According to the fractionation hypothesis^23, 24^, the low molecular weight (LMW) PCBs are able to travel farther in air and become relatively enriched farther away from their source regions in comparison to the high molecular weight (HMW) PCBs because they are more volatile and less associated with depositing particles^22^. The more volatile PCBs are also more likely to travel in the atmosphere and undergo “grasshopping” or to be re-emitted to the air from surface soils, a phenomenon called “secondary emission”. Once deposited, they are less strongly retained or absorbed onto surface soils and other condensed phases compared with the HMW PCB congeners^22^.

The LMW PCB congeners that are volatile and mobile in air in warmer climates are much less volatile in cold climates^22^. Modeling studies have also discussed the relative importance of secondary emissions vs. primary emissions of PCBs and have indicated that the emissions of lighter PCB28 are more likely dominated by secondary emissions rather than primary emissions compared with HMW PCBs (such as CB153 and CB180)^28–30^. Therefore, PCB28 was selected as the targeted compound in this study because it has more active physico-chemical properties than HMW PCB congeners, such as octanol-air partition coefficient, vapor pressure etc.

PCBs were manufactured in China from 1965 to 1974. During this period, approximately 1.0 × 10^4^ t of PCBs were produced (0.8% of the total global PCB production)^8^. The total emissions of PCB28 from intentional production (IP-PCB28), unintentional production (UP-PCB28) and e-waste were estimated in our previous study^31, 32^. The evaluated emission inventory of PCBs could serve as input data that drive models to study their environmental behavior and fate. For this reason, the main objectives of this study were (1) to assess the performance of the Chinese Gridded Industrial Pollutants Emission and Residue Model (ChnGIPERM), (2) to investigate the air-soil exchange behavior across China using the ChnGIPERM, (3) to explore the secondary emissions and residues in the non-source areas in China using the ChnGIPERM.

## Results and Discussion

### Characteristics of air-soil exchange

**Figure 1:**
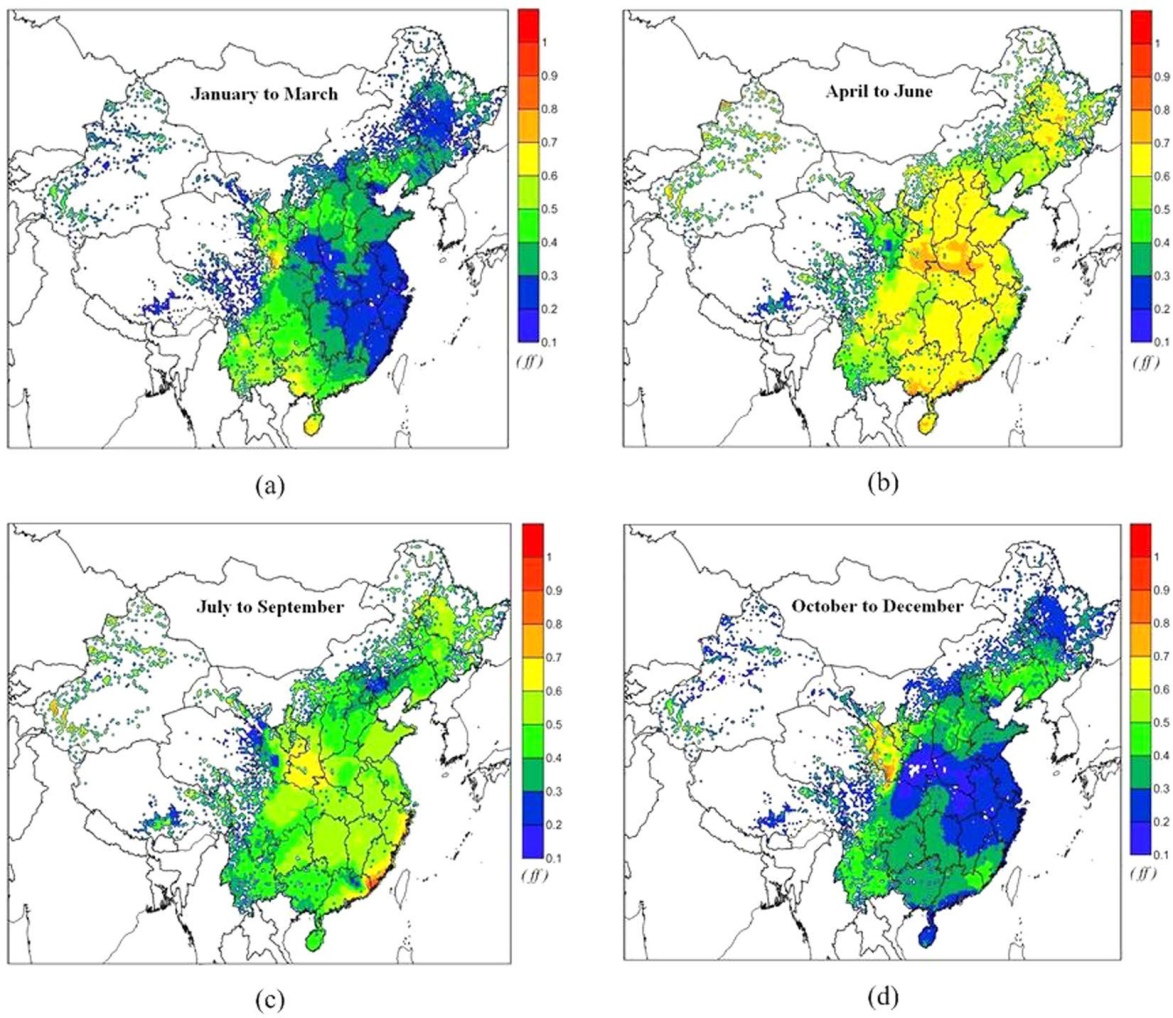
Temporal and spatial trends of air-soil exchange for PCB28 in 2005: (**a**) January to March, (**b**) April to June, (**c**) July to September, and (**d**) October to December. The maps were drawn by the software of Surfer 9.0, http://www.goldensoftware.com/.

PCB-containing products have mainly been applied in cities; therefore, the urban soil type (one of 6 soil types including urban land, dry cropland, paddy field, forest, grassland, and uncultivated land) was selected in the present study. The *f*_A_ and *f*_S_ in the fugacity fraction (*ff*) represent the first air layer and the first soil layer, respectively, which was employed to assess the air-soil exchange of PCB28 in 2005 (the numerical simulations were performed successively from 1965 to 2010 at a time step of one day). Figure 1 clearly depicts a net deposition (*ff* < 0.5) from air to soil in the majority of regions in China from January to March and October to December in 2005. In contrast, net volatilization (*ff* > 0.5) from soil to air occurred in most of the areas in China from April to June and July to September in the same year. Based on the modeled results, several factors affecting the variation characteristics of air-soil exchange are elaborated in the following sections.

### Influence of emission patterns and long-range atmospheric transport (LRAT)

**Figure 2:**
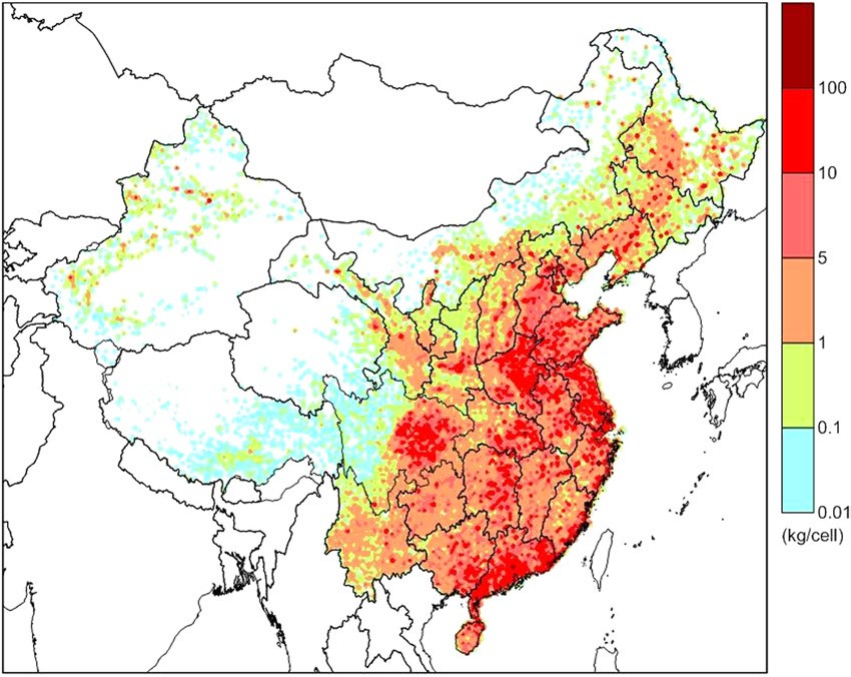
Gridded emission patterns in China from 1950 to 2010 at a resolution of 1/6° latitude × 1/4° longitude32. The maps were drawn by the software of Surfer 9.0, http://www.goldensoftware.com/.

According to the emissions inventory of PCB28 in China^32^ (Fig. 2), the most intensive emissions of PCB28 to air occurred in eastern, central west and southern China, including Beijing, Hebei, Henan, Shandong, Jiangsu, Shanghai, Chongqing and Guangdong provinces. Previous investigations have revealed that the major source regions, depending on the emissions pattern of the chemical, are characterized by high concentrations of PCB28 in air^33, 34^.

**Figure 3:**
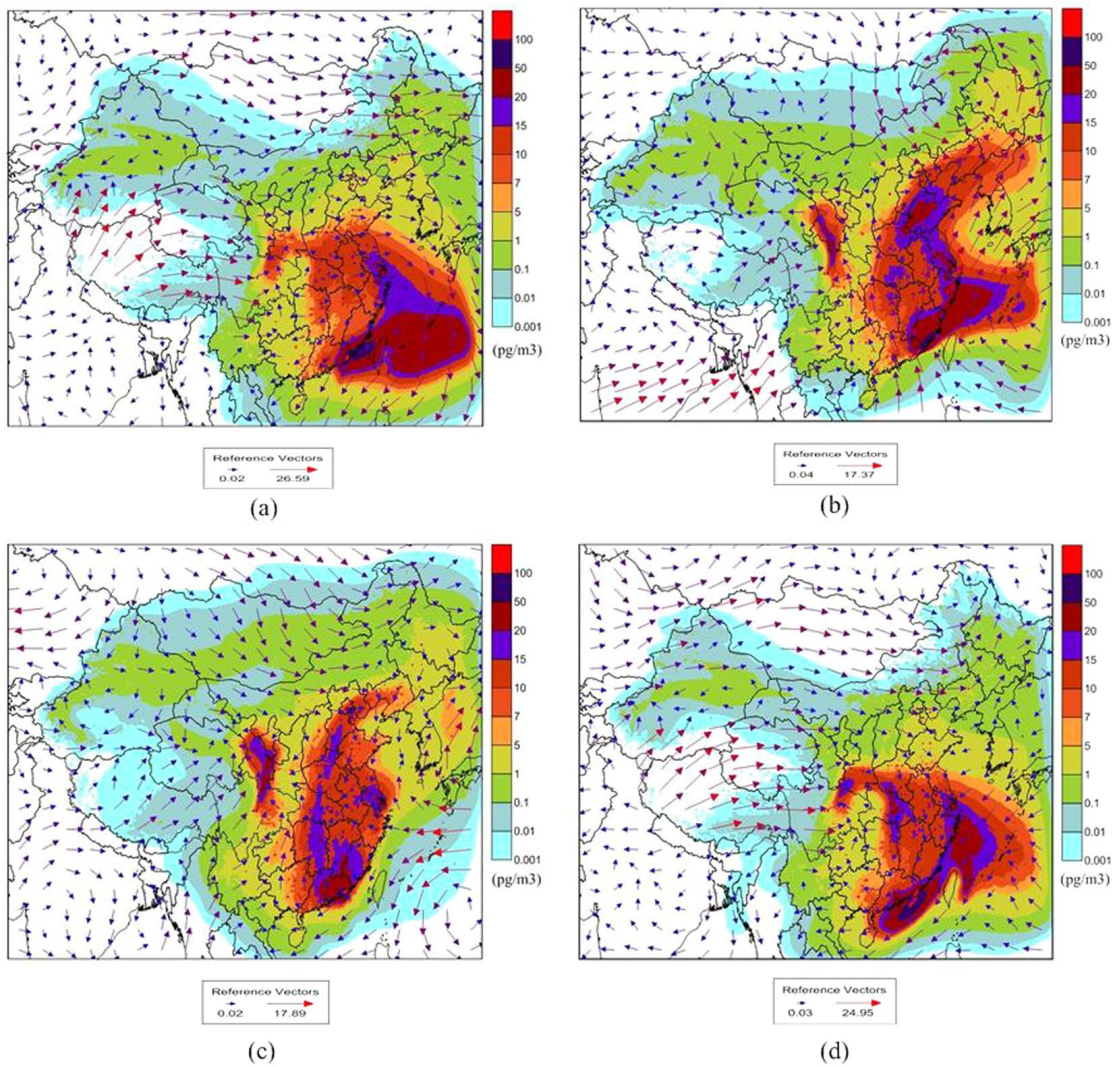
Air concentration of PCB28 in 2005: (**a**) January to March, (**b**) April to June, (**c**) July to September, and (**d**) October to December. The maps were drawn by the software of Surfer 9.0, http://www.goldensoftware.com/.

Figure 1(a and d) show the primary emissions pattern in the lower temperature seasons, indicating the net deposition in major areas in China especially in eastern and central regions, which is due to the higher air concentrations in these regions than other areas during these seasons (see Fig. 3). LRAT can carry pollutants from their sources to their sinks, which is an important factor affecting the distribution of chemicals in the environment. Generally, meteorological conditions such as the average of wind direction and speed of a day affect the transport direction and distance of pollutants. Figure 3 depicts the spatial distribution of the air concentration of PCB28, for which the transport pathway was obviously subject to the wind direction. On the other hand, the emissions patterns are in accordance with the air-soil exchange in the same period. The significant reductions in the air concentration from summer to winter in most areas in China imply a temperature dependence of the air-soil exchange (from volatilization to deposition). The different areas of high air concentrations (mass centers) of PCB28 were mainly subject to the emissions patterns and LRAT (in Fig. 3), and these areas subsequently strongly affected the air-soil exchange patterns of PCB28, especially in the low temperature seasons.

### Influence of soil organic carbon (SOC) and vertical transfer

Once POPs are released to the environment, the soil plays an important role and controls the mass balance because of the strong affinity of POPs for SOM^19^. SOC (the soil organic carbon (SOC) content can be estimated as 0.56 of the SOM) affects the accumulation of chemicals in soil. High spatial variability of PCB concentrations (up to four orders of magnitude) was found between soils in the UK and Norway and in undisturbed global background surface soils, suggesting the importance of SOC in the spatial distribution of these chemicals in surface soil. It has been observed that the correlation between soil concentrations of PCBs and soil SOC was statistically significant and strongest for the lighter PCBs^35^. A statistically significant relationship between soil concentrations of all PCB congeners and SOC contents was determined in the surface layers (0–1 cm) but not in the deeper layers (4–5 cm, 9–10 cm)^36^. In 1998 and 2008, grasslands and woodlands were sampled from 0 to 10 cm and split into surface (0~5 cm) and subsurface (5~10 cm) layers by Schuster and co-workers^37^. They found that higher amounts of PCBs generally appeared in the top layers in 1998; however, more sites had higher PCB loadings in the deeper grassland layers in 2008, while the top woodland layers were still higher in that same year.

In a previous study, we investigated the influence of SOM on residue concentrations of PCB congeners in soil in China^38^. There were significant positive correlations between the organic matter and the residue amount for all of the modeled PCB congeners in the first soil layer (depth of 0.1 cm) and for all of the modeled PCB congeners (except HMW PCB180) in the second soil layer (depth of 1 cm), but such correlation was negative in the third soil layer (depth of 20 cm), indicating that organic matter has an important influence on the distribution of PCBs in soil.

**Figure 4:**
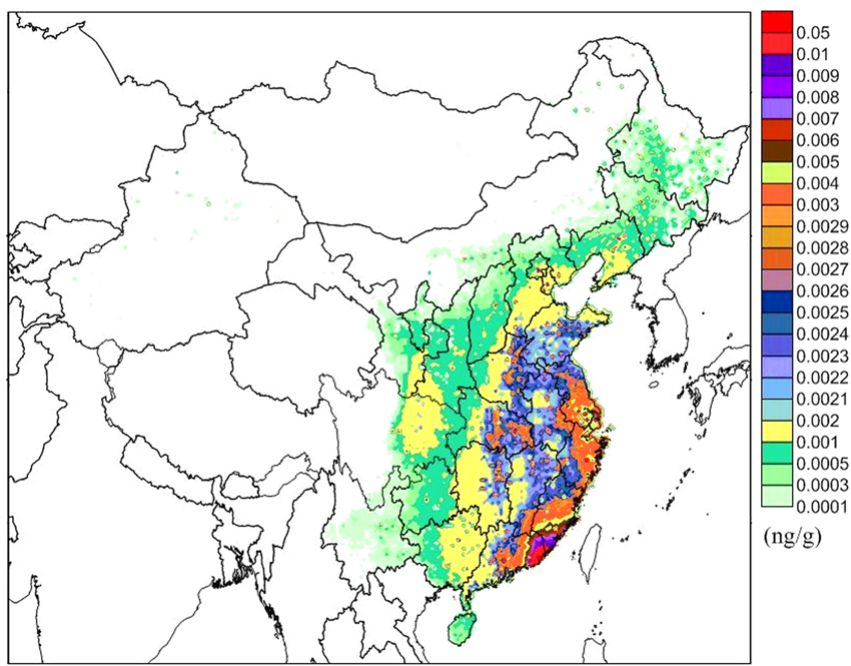
The concentration in the second soil layer in 2005. The map was drawn by the software of Surfer 9.0, http://www.goldensoftware.com/.

Once deposited to surface soil, the contaminants will move into deeper soil layers^36^, in order to further investigate the vertical transfer of contaminants in soil, the leaching potential (*L*p) was used to assess the leaching capacity from the top to deeper soil^39^. *L*p can be defined as

1 $${L}_{P}=S/({K}_{{\rm{OC}}}\cdot {V}_{P})$$

where *S* is the solubility of the chemical, in g·m^−3^; *K*_OC_ is the partition coefficient between soil and water, dimensionless parameter; and *V*_P_ is the vapor pressure of the chemical, in the unit of Pa. The leaching potentials (*L*_P_, at a reference temperature of 25 °C) of PCB28, PCB101, PCB153 and PCB180 were 2.47 × 10^−4^, 3.10 × 10^−6^, 4.40 × 10^−7^, and 1.65 × 10^−7^, respectively, indicating that the LMW PCB congeners could be more easily transferred to deeper soil layers than the HMW PCB congeners^38^. Figure 4 shows that the soil concentrations in the second soil layer for PCB28 in 2005 are high in eastern and central China. Compared with the air-soil exchange patterns (Fig. 1), there are reversals between the fugacity fraction *ff* and the soil concentrations in second soil layer. In particular, the concentrations in the second soil layer were high when the *ff* was relatively low in some regions; the vertical transfer of the PCB28 may reduce the soil concentration in the first soil layer, which directly affected the air-soil exchange of PCB28.

### Influence of temperature and precipitation

**Figure 5:**
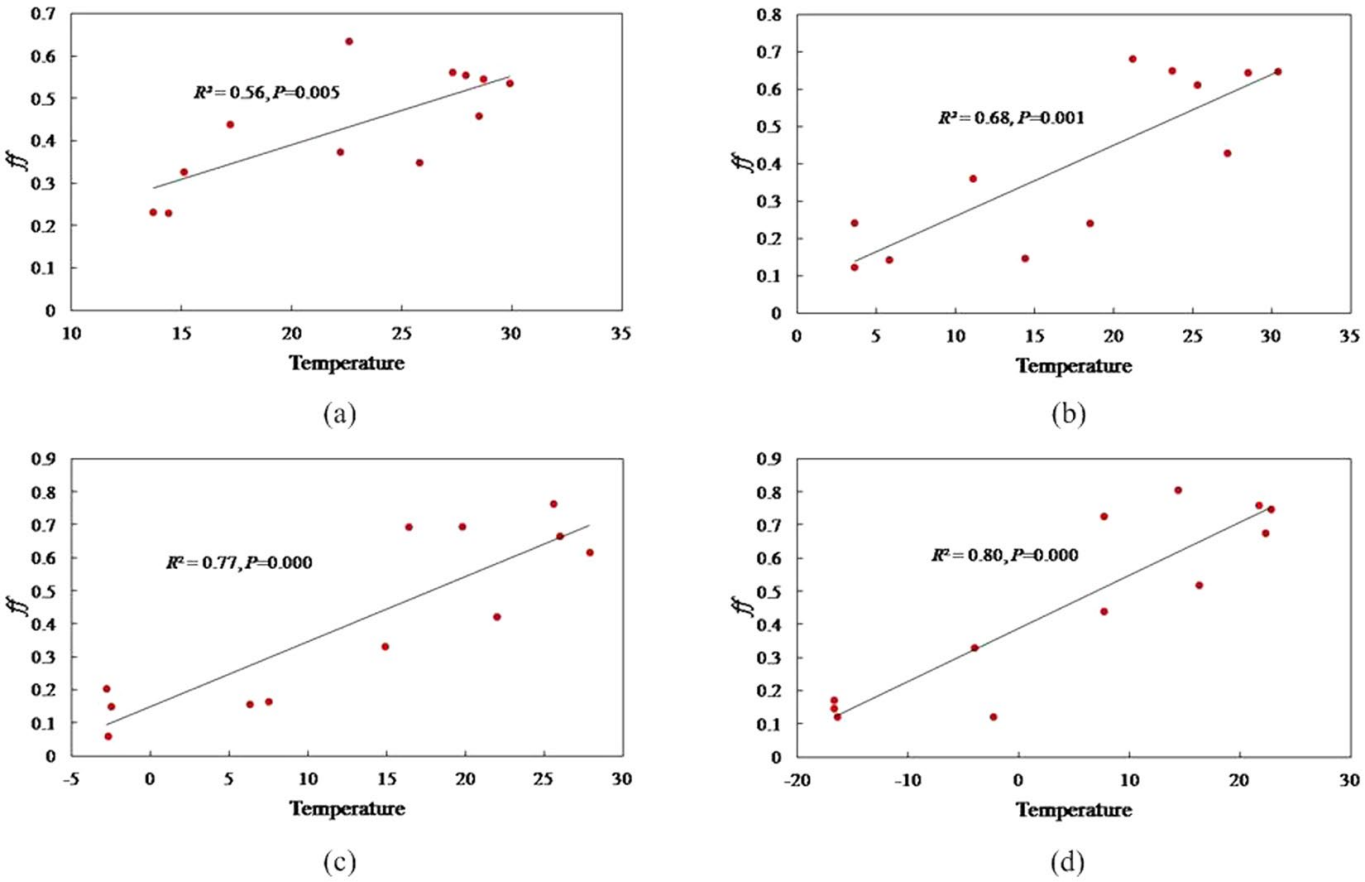
The relationships between the temperature and fugacity fraction (ff) for PCB28 in the latitudinal direction from south to north around four cities, (**a**) Guangzhou, (**b**) Wuhan, (**c**) Beijing, and (**d**) Harbin.

PCBs can also be re-emitted to the atmosphere after deposition to the surface soil due to temperature-dependent volatility, and this subsequent temperature-dependent re-volatilization from surface soil contributes significantly to their atmospheric concentration^40^. Figure 5 shows the region of latitudinal direction from ~22.2°N to ~46.4°N bounded by Guangzhou in the south and by Harbin in the north, which contains four cities, Guangzhou, Wuhan, Beijing, and Harbin from south to north, respectively (Fig. A8). The *ff* values and monthly average temperatures are significantly correlated in all cities in 2005. Notably, the corresponding temperature when *ff* = 0.5 at equilibrium decreases with an increasing temperature difference from low to high latitudes.

**Figure 6:**
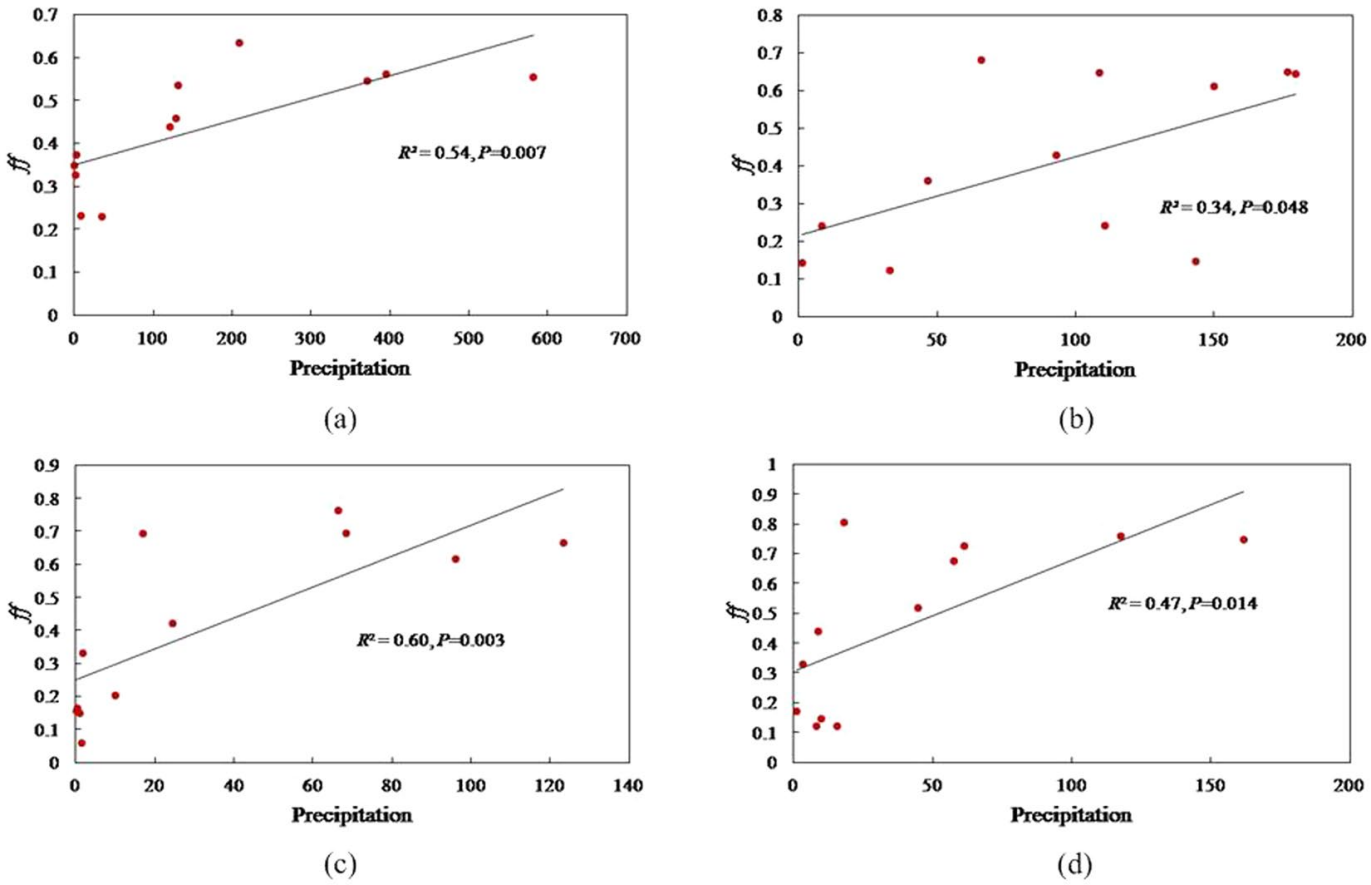
The relationships between precipitation and the fugacity fraction (ff) for PCB28 in the latitudinal direction from south to north with around four cities, (**a**) Guangzhou, (**b**) Wuhan, (**c**) Beijing, and (**d**) Harbin.

Precipitation scavenging is also an important factor in establishing the LRAT potential for some chemicals. Particles with adsorbed chemicals may be scavenged or swept out of the air by wet deposition with raindrops or snow. Thus, precipitation has the potential to remove a considerable quantity of aerosols from the atmosphere. Rain or snow is therefore often highly contaminated with chemicals such as PCBs^19^. Similar to temperature, the *ff* was also significantly correlated with the monthly average precipitation in 2005 in the same cities (Fig. 6). The *ff* increased with precipitation enrichment, which implies that precipitation can effectively scavenge PCB28 from air, causing the fugacity in air to decrease.

Both temperature and precipitation affect the partitioning of chemicals between air and soil, but the magnitude of the change in raising temperature (favoring the gas phase) is more influential than increased precipitation rates (favoring air-to-surface soil deposition) in determining the overall environmental fate and air-soil exchange of such chemicals^41^. In fact, the temperature and precipitation affect chemicals through different processes in the environment. Temperature can affect the presence of a chemical in the environment in a bidirectional manner. Low temperatures result in condensation that can cause deposition of chemicals in cold regions, and high temperatures result in the volatilization of chemicals from surface soils. However, precipitation has a unidirectional impact on chemicals in the environment by scavenging chemicals from the air and brings them to surface soils.

### Spatial distribution patterns of emissions and residues

**Figure 7:**
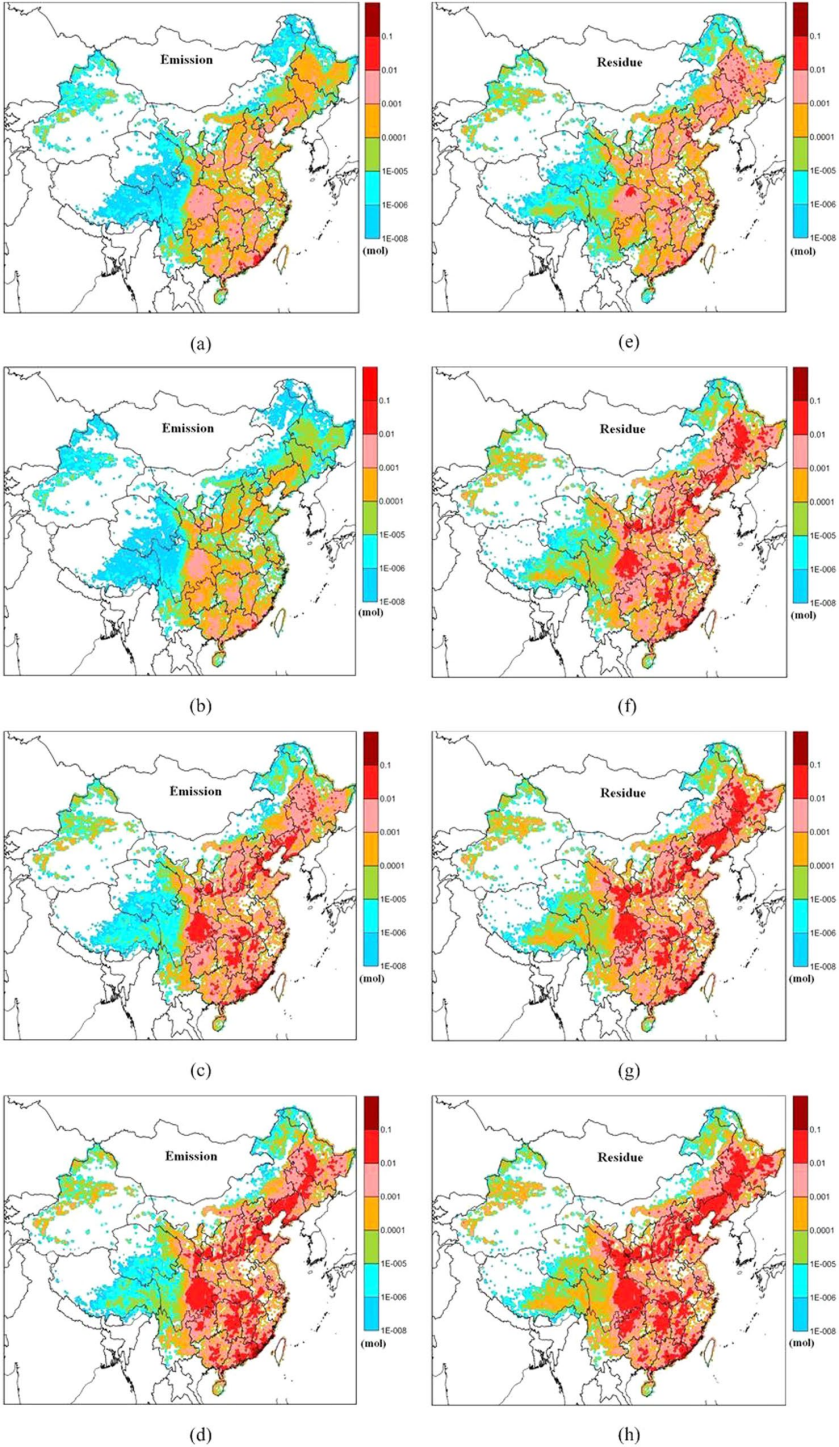
Spatial distribution patterns of cumulative emissions and residues for PCB28: (**a**) and (**e**) January; (**b** and **f**) April; (**c** and **g**) July; and (**d** and **h**) October in 2005 since 1965. The maps were drawn by the software of Surfer 9.0, http://www.goldensoftware.com/.

The spatial distribution patterns of the cumulative emissions and residues of PCB28 in January, April, July, and October, 2005 since 1965 are presented in Fig. 7. In general, the emissions and residues gradually increased with time, and the accumulated residues were higher than the accumulated emissions in most regions of China, especially in the relatively colder regions such as northeastern China, western Sichuan province, northern Xinjiang province, Qinghai-Tibetan Plateau, and northeastern Inner Mongolia.

Clearly, the soil residues in the Qinghai-Tibetan Plateau were higher than the emissions by approximately two orders of magnitude. This region has a relatively low population density and less usage of PCB-containing products but contains high residues relative to the emissions. Perhaps the LRAT, low temperature, and secondary fractionation affect the distribution patterns of the emissions and residues. In fact, secondary sources begin to play a more important role, and PCBs can more freely exchange between the air and soil and become subject to “grasshopping”, which is termed “secondary fractionation”^22^. Secondary fractionation, however, drives the longer-term accumulation of persistent chemicals in cold climates, especially LMW PCBs.

### Secondary emissions and residues

In order to investigate the characteristics of secondary emissions and residues of PCB28 in soil in non-source areas, which did not have direct input of PCB28 and the occurrence of PCB28 in soils were mainly from the atmospheric deposition of this compound through atmospheric transport from source areas, we define emission factor *EF* of PCB28 from secondary residues as

2 $$EF={\rm{secondary}}\,\mathrm{emissions}/\mathrm{secondary}\,{\rm{residues}}$$

Four sites in the non-source areas, including G1(183, 38), G2(172, 85), G3(160, 135), and G4(217, 202) in the order from low latitude to high latitude, were selected (in Fig. A8,SI), and the *EF* of PCB28 at these 4 sites are depicted in Fig. A10(SI).

As shown in Fig. A10(SI), the values of EF gradually decrease from low to high latitude, or from high to low temperature, and the mean value of 8.36 × 10^−6^ for G1, 4.77 × 10^−6^ for G2, 1.68 × 10^−6^ for G3 and 7.50 × 10^−7^ for G4, respectively. The *EF* values reached almost zero for Sites G3 and G4 in cold months, November, December, January, and February, while those at Sites G1 and G2 were still quite high.

**Figure 8:**
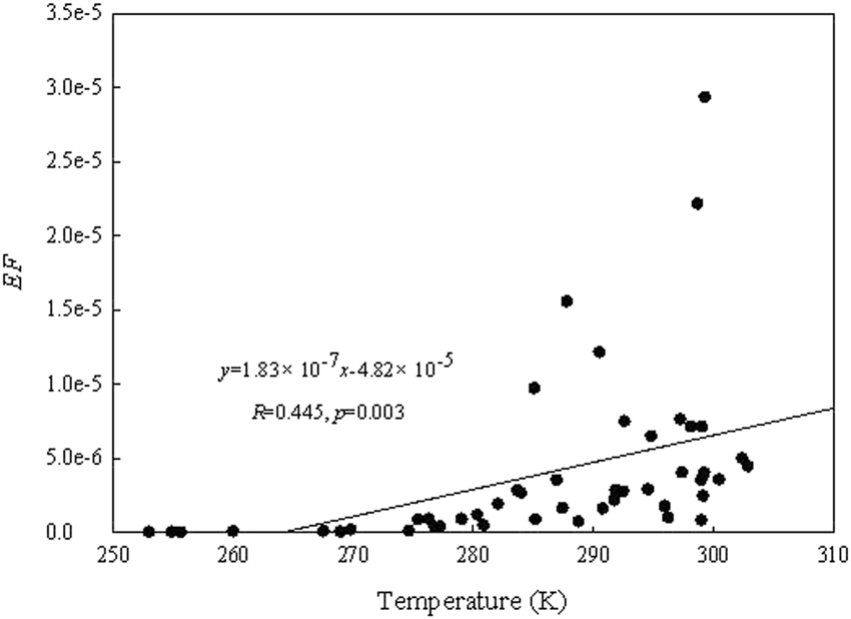
The relationship between emission factors (EF) and temperature.

The main driving force for the different secondary emission ability of PCB28 at the 4 sites is actually the temperature. Figure 8 presents the relationship between *EF* and temperature, showing that the values of *EF* of PCB28 is significantly correlated with the temperature (*R* = 0.445, *p* = 0.003), which suggests that the temperature is an important factor to affect the emission characteristic of PCB28.

Before the application of PCB28, the secondary residues of PCB28 in soil at these sites were equal to zero. As soon as PCB28 was used, this chemical started to emit to air, and the atmospheric transport occurred. Almost simultaneously, PCB28 started to accumulate in soil from atmospheric depositions due to long- and short-range atmospheric transport of PCB28 from the primary sources, obviously showing a primary distribution pattern, the closer to the sources, the higher of the concentrations in soil. However, due to higher *EF* in warm regions than in cold regions, PCB28 emitted in the warm regions (such as Sites G1 and G2) could end up in the cold regions (such as Sites G3 and G4), gradually forming the secondary distribution pattern, the concentrations in the colder regions were higher than those in the warmer regions.

## Conclusions

The ChnGIPERM was established in this study to simulate the environmental behavior and fate of industrial organic pollutants, and the model’s performance was examined and evaluated. Using the ChnGIPERM, modeling investigations were carried out to evaluate the environmental fate, behavior, and distribution of PCB28 in the environment from 1965 to 2010 in China. We investigated several factors that impact the air-soil exchange, distribution patterns and characteristics of secondary emissions and residues in the non-source areas. The emission patterns, atmospheric transport, SOC, soil vertical transfer, ambient temperature, and precipitation were important factors. Hence, the ChnGIPERM can be used to further investigate the environmental behavior and fate and source-sink analysis of other industrial POPs.

## Methods

### Model description

The ChnGIPERM in this investigation was developed based on the Chinese Gridded Pesticide Emission and Residue Model (ChnGPERM) and the Gridded Basin-based Pesticide Mass Balance Model (GB-PMBM). The ChnGPERM has used in numerical studies of the α-HCH budget and atmospheric outflow from China and the environmental fate of β-HCH^33, 42^. The GB-PMBM was also applied to assess the α-HCH budget in the Taihu region, China^43^. The ChnGIPERM has some differences from both ChnGPERM and GB-PMBM, such as input mode of PCB28 inventory, transport module, soil types, dry deposition and some parameters with dependent temperature etc. ChnGIPERM is a gridded mass balance model based on a gridded system with a resolution of 1/6° latitude and 1/4° longitude. The main body of the model includes both transfer and transport modules. For the transfer module, a level IV fugacity method was employed to calculate and describe the changes in industrial pollutant concentrations and inter-compartmental transfer of the modeled chemicals in the multimedia environment. The transfer module consists of 6 soil types (urban land, dry cropland, paddy field, forest, grassland, and uncultivated land) in 4 vertical soil layers; a water compartment; a sediment compartment; and an air compartment that includes two layers, the atmospheric boundary layer (ABL, 0~1000 m) and the atmospheric low troposphere (ALT, 1000~4000 m). The transport module describes the mass exchange of chemicals between the simulation grid cells driven by atmospheric transport (wind directions and speed) and water currents.

In this study, the ChnGIPERM domain covers the whole country of China, spanning from 17° to 55°N and from 70° to 135°E. A detailed description of the structures of the model can be found in the Supplementary Information (SI) of this paper.

### Historical PCB emissions and other input data

A gridded PCB28 emission inventory with a resolution of 1/6° latitude and 1/4° longitude was applied as the basic input data for the model operation. In our previous study, we compiled a comprehensive emission inventory for PCB28 that includes IP-PCB28 (1965 to 2010), UP-PCB28 (1950 to 2010), and e-waste PCB28 (1990 to 2010). The model was integrated from 1965 because UP-PCB28 had lower emissions than the other emission modes. The transport of water currents is neglected because most of the underlying surfaces over the model domain are covered by land.

The model inputs also include gridded daily meteorological data (temperature, precipitation, wind direction and speed for different elevations), soil characteristic parameters (density, organic carbon content and porosity), surface features (urban, dry cropland, paddy field, grass, forest, uncultivated land and water types), and physico-chemical properties of the chemical. These details are also presented in the SI.

Due to the limited monitoring data for PCBs across China, which was used to assess the reliability of the model, and the modeled results can reflect the environmental distribution characteristics of chemical. Therefore, monitoring data were obtained for 2004^44^, 2005^10^, and 2008^45^ across China, and the numerical simulations were performed successively from 1965 to 2010 at a time step of one day.

### Model evaluation

The monitoring data are collected from two sources. (1) The Chinese POPs Soil and Air Monitoring Program (SAMP-I) operated by the International Joint Research Center for Persistent Toxic Pollutants (IJRC-PTS) archived soil and air samples across China from June 2005 to June 2006, which were analyzed in the laboratories of IJRC-PTS^9, 10^. (2) The other data are from two studies in the literature. First, Jaward deployed air-monitoring samplers in 2004, in both rural and urban settings (13 rural and 19 urban sites) in China^44^. Second, Hogarh deployed air-monitoring samplers in 2008, in both rural (3 sites) and urban (17 sites) in China^45^.

According to the time sequence, the modeled data in this study compared with the different monitoring data, as follows. (1) The modeled air concentrations were compared with the measured air concentrations from Jaward *et al.* ^44^ for PCB28 in 2004 by calculating the Pearson correlations between the modeled and measured air concentrations at the 30 sites (12 rural and 18 urban, except Hong Kong) at the corresponding grid cells where the sampling sites were located at the same sampling time: *R* = 0.55, *P* = 0.002, for all sites; *R* = 0.66, *P* = 0.021, for rural sites; and *R* = 0.78, *P* = 0.000, for urban sites (except CU-17 because the modeled air concentration was relatively low). (2) The monitoring data from IJRC-PTS^10^ was compared with the modeled mean soil concentrations for PCB28. The Pearson correlations were calculated between the measured and modeled soil and air concentrations across China, except the individual data for which the modeled values were relatively low in rural and background areas, and the results of comparison are *R* = 0.49, *P* = 0.001, for soil and *R* = 0.26, *P* < 0.05, for air. (3) In comparing the monitoring data from Hogarh *et al.* ^45^, the rural sites were neglected because of inadequate data. The air concentrations of trichlorobiphenyl and modeled PCB28 were available at fourteen urban sites (except Hong Kong). The Pearson correlations between the measured and modeled air concentrations were *R* = 0.70, *P* = 0.005 for the urban sites. The comparison between the modeled data and all of the measured data suggests that the modeled results agree reasonably well with the monitoring data for air and soil of China.

To gain further insight into the model’s performance, the overall mass balance of PCB28 in the model domain from 1965 to 2010 is estimated to assess the mass conservation of the model. The small relative error (<0.1%) between the total input and output masses proves that the model achieves excellent mass conservation.

## Electronic supplementary material

Supplementary Information
