# Supplementary material for: Modeling the air-soil exchange, secondary emissions and residues in soil of polychlorinated biphenyls in China
Source: Sci Rep. 2017 Mar 16;7:221. doi: 10.1038/s41598-017-00351-0 (PMC5428302; doi:10.1038/s41598-017-00351-0)
Supplement: Supplementary file 1 — Supplementary Information [file 41598_2017_351_MOESM1_ESM.doc]

**IJRC-PTS**

**Supporting Information (SI) for**

**Modeling the air-soil exchange, secondary emissions and residues in soil of polychlorinated biphenyls in China**

Authors: Song Cui^1,2^, Qiang Fu^1^, Yi-Fan Li^2,3,1*^, Jianmin Ma^4^, Chongguo Tian^5^ , Liyan Liu^2^& Leiming Zhang^6^

Affiliations:

^1^ International Joint Research Center for Persistent Toxic Substances (IJRC-PTS), School of Water Conservancy and Civil Engineering, Northeast Agricultural University, Harbin, Heilongjiang, 150030, China

^2^ IJRC-PTS, State Key Laboratory of Urban Water Resource and Environment, Harbin Institute of Technology, Harbin, Heilongjiang, 150090, China

^3^ IJRC-PTS-NA, Toronto, M2N 6X9, Canada

^4^ Key Laboratory of Western China’s Environmental System, Ministry of Education, College of Earth and Environment Sciences, Lanzhou University, Lanzhou, Gansu, 730000, China

^5^ Yantai Institute of Coastal Zone Research, Chinese Academy of Sciences, Yantai, Shandong, 264003, China

^6^ Air Quality Research Division, Science and Technology Branch, Environment Canada, 4905 Dufferin Street, Toronto, Ontario, M3H 5T4, Canada

* Corresponding author: Yi-Fan Li

E-mail address: ijrc_pts_hit06@yahoo.com

Number of pages: 24

Number of tables: 3

Number of figures: 10

**Contents**

A1. Model region and grid system·················································································2

A2. Model framework····································································································2

A 2.1 Transfer module·····························································································4

A 2.2 Transport module·························································································11

A3. Model input data····································································································13

A4. Model evaluation···································································································17

A 4.1 Comparison between modeled and measured soil concentrations···············17

A 4.2 Comparison between modeled and measured air concentrations················18

A5. Air-soil exchange and secondary emissions and residues·····································19

A5.1 Air-soil exchange·································································································19

A5.2 Secondary emissions and residues·······································································21

References····················································································································22

**1**

# A1. Model region and grid system

Chinese Gridded Industrial Pollutants Emission and Residue Model (ChnGIPERM) used in this study has been developed to describe transport, transfer, and environmental behavior of PCB28 in China from 1965 to 2010. Model domain and grids adopted in the present study are illustrated in Fig.A1.


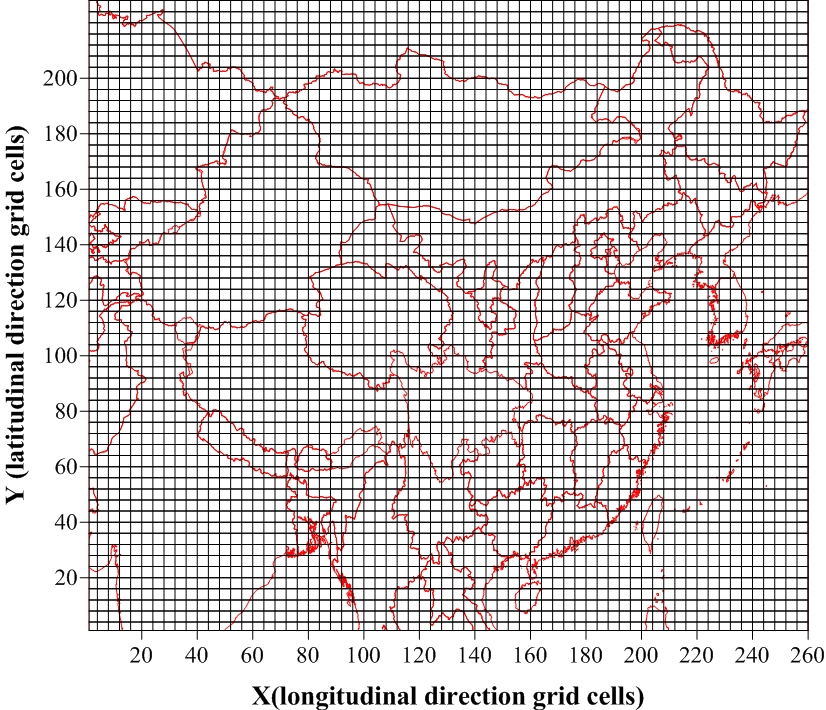


### Figure A1. Model domain and grid system (1 cell=1/4°×1/6° latitude/longitude, 1 cell = approximately 24×24 km). The map was drawn by the software of Surfer 9.0, http://www.goldensoftware.com/.

# A2. Model framework

The ChnGIPERM employed in this investigation is developed based on both Chinese Gridded Pesticide Emission and Residue Model (ChnGPERM), which has been used in numerical studies of α-HCH budget and atmospheric outflow from China and environmental fate of β-HCH[1, 2], and Gridded Basin-based Pesticide Mass Balance Model (GB-PMBM), which has been applied to assess α-HCH budget in the Taihu region, China [3]. ChnGIPERM is a gridded mass balance model on a gridded system with a 1/6° latitude and 1/4° longitude resolution.

**2**

### Figure A2. The modeling processes in a grid cell. The map was drawn by the software of Microsoft Visio 2010, https://products.office.com/.

The main body of the model that including both transfer and transport modules. For the transfer module, a level Ⅳ fugacity method was employed to calculate and describe the changes in industrial pollutant concentrations and inter-compartmental transfer of the modeled chemicals in the multimedia environment. The transfer module consists of 6 soil types in 4 soil vertical layers, water, sediment, and the air compartment that including two layers are the atmospheric boundary (ABL, 0~1000 m) and the atmospheric low troposphere (ALT, 1000~4000 m). There are six soil types, including urban land, dry cropland, paddy field, forestry, grassland, and uncultured land. In the soil matrixes, the model adopts four well-mixed soil layers, with depths at 0.1cm, 1.0 cm, 20 cm, and 30 cm from top to bottom [4].The transport module describes the mass exchange of chemicals between simulation grid cells driven by atmospheric transport (wind direction and speed) and water current. Detailed transfer and transport processes considered in the model at a model grid cell are displayed in Fig.A2.

**3**

# A2.1 Transfer module

A dynamic fugacity theory was employed to calculate the concentrations and inter-compartmental transfer of the PCB28 between adjacent compartments at a grid cell [5]. For each compartment at a grid cell, the change rate of fugacity equals to the difference between input and output rates divided by a product of compartment volumes and fugacity capacity. The following differential equations (A1-7) describe the change in fugacity in four soil layers with various soil types, air, water, and sediment as:

(A1)

(A2)

$\begin{matrix} V_{1,III}Z_{1,III}df_{1,III}/dt=(D_{II-III,v}+D_{II-III,l})f_{1,II}+D_{IV-III,v}f_{1,IV} \\ -(D_{III-II,v}+D_{III-IV,v}+D_{III-IV,l}+D_{III-0,r})f_{1,III} \end{matrix}$ (A3)

$V_{1,IV}Z_{1,IV}df_{1,IV}/dt=(D_{III-IV,v}+D_{III-IV,l})f_{1,III}-(D_{IV-III,v}+D_{IV-0,l}+D_{IV-0,r})f_{1,IV}$ (A4)

(A5)

$\begin{matrix} V_{3}Z_{3}df_{3}/dt=T_{0-3,a}+(D_{2-3,v}+D_{2-3,q}+D_{2-3,d}+D_{2-3,m})f_{2}+(D_{4-3,v}+D_{4-3,b})f_{4} \\ -(D_{3-2,v}+D_{3-4,v}+D_{3-4,s}+D_{3-0,r})f_{3} \end{matrix}$ (A6)

$V_{4}Z_{4}df_{4}/dt=(D_{3-4,v}+D_{3-4,s})f_{3}-(D_{4-3,v}+D_{4-3,b}+D_{4-0,r})f_{4}$ (A7)

In Eqs (A1-7), T and D represent quantity of transfer and transfer rate coefficient, f, V, Z and t are fugacity, compartment volume, fugacity capacity and time, respectively. The subscript 1, 2, 3 and 4 represent each compartment in the sequence of soil, air, water, and sediment, respectively. The subscript I, II, III and IV are four soil layers from the upper to bottom, respectively. Subscript v, q, d, m, l, r, b, s, a, and e represent the diffusion (absorption and volatilization), dry deposition, wet deposition, rain dissolution, leaching, degradation, resuspension, sedimentation, air advection, and emission data of chemical, respectively.

**4**

The calculation of partition coefficients, Z and D in non-diffusive processes are outlined in Table A1 according to previous studies [4, 5]. The values of corresponding environmental parameters to calculate the Z values in Table A1 and corresponding physicochemical properties of PCB28 used in the model are presented in Table A2.

**Table A1** Calculation of partition coefficients, Z values

| **Partition coefficients** | Equation |
| --- | --- |
| Octanol-air | log Koa = a + b×*RRT*, a=-2.0296+1310.7/T, b = -5.5305+5879.8/T |
| Organic matter-air | K_om-a_=K_oa_ |
| Soil-air | K_sa_=Z_s_/Z_a_ |
| Aerosol-air (m^3^/μg) | logK_P_=logK_oa_+logф_aom_-11.91 |
| Aerosol-air (dimensionless) | K'_P_=K_P_T_SP_=Z'_q_/Z_a_ |
| Air-water | K_aw_= K_H_(T)/RT^*^ |
| Mineral matter-water | logK_mw_=0.7logA_W_-10.68 |
| Mineral matter-air | K_ma_=K_mw_K_aw_ |
| Suspension-water (dimensionless) | K'_sus_=ф_soc_K_oc_ρ_sus_ |
| Sediment-water(dimensionless) | K_se_=ф_seoc_K_oc_ρ_se_ |
| **Z values** (mol m^-3^ Pa^-1^) |  |
| Air | Z_a_=1/RT |
| Organic matter | Z_om_=Z_a_K_oa_ |
| Aerosol | Z'_q_=K'_p_Z_a_ |
| Water | Z_w_=1/K_H_(T) |
| Mineral matter | Z_mm_=K_mw_Z_w_ |
| Suspension | Z_sus_=Z_w_K'_sus_ |
| Sediment | Z_se_=Z_w_K'_se_ |
| Bulk soil | Z_1_=ф_sa_Z_a_+ф_sw_Z_w_+ф_som_Z_om_+ф_smm_Z_mm_ |
| Bulk air | Z_2_=Z_a_+Z'_q_ |
| Bulk water | Z_3_=Z_w_+Z_sus_ |
| Bulk sediment | Z_4_=ф_se-w_Zw+(1-ф_se-w_)Z_se_ |

^*^ log(K_H_(T))=11.97-3100/T [7]; R=8.314 Pa m^3^ mol^-1^K^-1^; T is absolute temperature.

**Table A2** The physico-chemical properties parameters of the PCB28 used in the model

| Parameters(unit) | Value | Ref |
| --- | --- | --- |
| Molar mass (g mol^-1^) | 257.54 | [6] |
| Molar volume:V_M_ (cm^3^ mol^-1^) | 289.1 | [6] |
| Henry’s law constant (Pa m^3^ mol^-1^ )(25^0^C) | 31 | [6] |
| Melting point (^0^C) | 57 | [6] |
| Aqueous solubility (g m^-3^)  Vapor pressure (Pa)  **5** | 0.16  0.034 | [6]  [6] |
| Continuing tableA2 | | |
| Parameters(unit) | Value | Ref |
| Log Koc (dimensionless) (25^0^C) | 4.28 | [6] |
| Log Kow (dimensionless) (25^0^C) | 5.67 | [6] |
| Degradation rate in air: K_a_ (h^-1^) | 3.50E-7 | [7] |
| Degradation rate in water: K_rw_ (h^-1^) | 4.07E-5 | [8] |
| Degradation rate in sediment: K_rse_ (h^-1^) | 1.26E-5 | [8] |
| Base degradation rate in the six kinds of soil: K_s0_ (h^-1^) | 1.26E-5 | [8] |
| Activation energy of deg. by OH radicals (J mol^-1^) | 8314 | [7] |
| Activation energy for freshwater (J mol^-1^) | 30000 | [9] |
| Activation energy for coastal/oceanic water (J mol^-1^) | 30000 | [9] |
| Activation energy for soil (J mol^-1^) | 30000 | [9] |

In the exchange layer, volatilization from the top soil layer to air is defined by

$D_{v}=\frac{1}{\frac{1}{D_{e}}+\frac{1}{D_{a}+D_{w}}}$, (A8)

where chemical diffuse in the boundary layer is given by

$D_{e}=A_{r}K_{v}Z_{a}$, (A9)

where A_r_ is the area of the model cell (24 km × 24 km). K_v_ is the boundary-layer mass transfer coefficient, and

$Z_{a}=\frac{1}{RT}$, (A10)

is the chemical capacity for air. Where the boundary-layer mass transfer coefficient can be expressed by

$K_{v}=\frac{B_{a}}{0.00475}$ (A11)

In which B_a_ is given in Eq (A14).

The chemical diffuses in air is defined by

$D_{a}=A_{r}B_{ae}\frac{Z_{a}}{Y_{d}}$, (A12)

where

**6**

$B_{ae}=\frac{B_{a}\varphi_{sa}^{10/3}}{(\varphi_{sa}+\varphi_{sw})^{2}}$ (A13)

in which the molecular diffusivity in air (m^2^ h^-1^) is expressed as

$B_{a}=\frac{0.186\times10^{0.0283T}}{V_{m}^{0.213}}$, (A14)

and φ_sa_ is volume fraction air in soil, φ_sw_ is volume fraction water in soil, and V_m_ is molar volume (cm^3^ mol^-1^).

The chemical diffuses in water is defined by

$D_{w}=A_{r}\frac{B_{we}Z_{w}}{Y_{d}}$, (A15)

where Y_d_ is the log mean diffusion distance in soil (layer 1, 2, 3, and 4), defined by Mackay [5]

$Y_{d}=\frac{dep_{bot}-dep_{top}}{\ln\left(dep_{bot}/dep_{top}\right)}$ (A17)

where dep_bot_ and dep_top_ are depth of bottom and top for soil layer, and

$B_{we}=\frac{B_{w}\varphi_{sw}^{10/3}}{(\varphi_{sa}+\varphi_{sw})^{2}}$ ,

In which the diffusivity in water (m^2^ s^-1^) can be expressed by

$B_{w}=\frac{4.864\times10^{-3}}{\mu^{0.905}V_{m}^{1.32}}$, (A18)

where μ is molecular viscosity in water (= 4.96).

In the exchange layer, the volatilization from the buffer layer is described by the diffusion between this layer and buffer layer. Generally, diffusions between soil layers n and n+1 are defined by

$\frac{1}{D_{vn,n+1}}=\frac{1}{D_{an}+D_{wn}}+\frac{1}{D_{an+1}+D_{wn+1}}$ , (A19)

**7**

where D_a_ and D_w_ are given by Eqs. (A12) and (A15). Other “D” values are given below.

The dry particle deposition is

$D_{q}=G_{q}Z_{q}$, (A20)

where G_q_ is the dry particle deposition flux,

$G_{q}=A_{r}v_{d}$, (A21)

v_d_ (m s^-1^) is the dry deposition velocity, which is calculated by the formulae based on [5] result:

$V_{\text{d}}(cm/s)=\left(0.04u_{*}^{2}+0.02\right)\cdot\left(Z_{0}/10^{-3}\right)^{0.33}$, (A22)

where *u** is friction velocity, *Z*_0_ (m) is roughness length, and

$Z_{q}=\frac{K_{p}T_{sp}}{RT}$. (A23)

In (A23) K_p_ is the particle-gas partition coefficient (m^3^ μg^-1^), given by [10]

$\log K_{p}=\log K_{oa}+\log\varphi_{aom}^{©}-11.91$, (A24)

where *φ’_aom_* is the mass fraction of organic carbon (=0.2), and *K*_oa_ is the octanol-air partition coefficient, given by

log *K*_oa_ = a + b×*RRT*, a = -2.0296+1310.7/T, b = -5.5305+5879.8/T (A25)

In the above equation, *RRT* is the coefficient (=0.3993, for PCB28) [10].

The predicted particulate fraction *φ* for chemical is calculated as

(A26)

where TSP (μg m^-3^) is the total suspended particle matter.

The diffusion due to wet particle deposition D_d_ is geiven by

$D_{d}=G_{d}Z_{q}$. (A27)

Z_q_ is computed from Eq. (A23), G_d_ is the wet deposition flux:

**8**

$G_{d}=G_{c}Q_{r}$, (A28)

where precipitation flux is defined by

$G_{c}=A_{r}V_{R}$, (A29)

where V_R_ is the precipitation rate (m s^-1^), and the aerosol scavenging ratio Q_r_ has a typical value of 4.0 × 10^5^ as given before.

The rain dissolution rate D_m_ is defined by

$D_{m}=G_{c}Z_{q}$, (A30)

where G_c_ and Z_q_ are given by Eqs. (A29) and (A23), respectively.

The leaching in soil D_l_ is defined by,

$D_{l}=G_{l}Z_{w}$, (A31)

where,

$G_{l}=A_{r}L_{w}$, L_w_ is the leaching rate (typical value = 0.002), and

$Z_{w}=\frac{1}{H}$. (A32)

Transfer rate coefficient of diffusions between water and air (D_2-3,v_ and D_3-2,v_) in Eqs (A5-6) is defined by

$D_{v}=A_{r}Z_{w}K_{OL}$ (A33)

where A_r_ is the area of water surface at a model grid cell. K_OL_ is the overall mass transfer coefficient is defined by Schwarzenbach [11]

$\frac{1}{K_{OL}}=\frac{1}{k_{w}}+\frac{Z_{w}}{Z_{a}k_{a}}$ (A34)

where k_w_ and k_a_ are water-side and air-side mass transfer coefficients, respectively. They are defined by

$k_{a}=10^{-3}+46.2\times10^{-3}U^{*}Sc(a)^{-0.67}$ (A35)

**9**

$\begin{matrix} k_{w}=10^{-6}+144\times10^{-4}(U^{*})^{2.2}Sc(w)^{-0.5}\text{ }(U^{*}<0.3ms^{-1}) \\ k_{w}=10^{-6}+34.1\times10^{-4}U^{*}Sc(w)^{-0.5}\text{ }(U^{*}>0.3ms^{-1}) \\ U^{*}=[(0.61+0.063U_{10})\times10^{-3}]^{0.5}U_{10} \end{matrix}$ (A36)

where Sc(a) and Sc(w) are the air (=2.9) and water (=1000) phase Schmidt number, U_10_ is the wind speed at 10 m height.

Transfer rate coefficient of diffusions between water and sediment (D_3-4,v_ and D_4-3,v_) in Eqs (A6-7) is described by

$\frac{1}{D_{v}}=\frac{1}{D_{w}}+\frac{1}{D_{se}}$ (A37)

The chemical diffuses in water (D_w_) and sediment (D_se_) are defined by

$D_{w}=A_{r}Z_{w}K_{w}$ (A38)

$D_{se}=A_{r}Z_{se}K_{se}$ (A39)

In Eqs (A37-38), the water-side boundary-layer mass transfer coefficient can be expressed by

$K_{w}=\frac{B_{w}}{Y_{w}}$ (A40)

where the diffusivity in water (B_w_) is given by Eq (A18) and Y_w_ is the boundary-layer thickness of water (0.0001 m).

The boundary-layer mass transfer coefficient of sediment-side is defined by

$K_{se}=\frac{B_{se}}{Y_{se}}$ (A41)

where B_se_ is the diffusivity in sediment and Y_se_ is the log mean diffusion distance in sediment which is solved by Eq (A17). B_se_ is given by Wu and Gschwend [12]:

(A42)

where φ_se-w_ is the volume fraction of water in sediment (=0.43) [1]. K_se_ is partition coefficients between sediment and water, expressed by

**10**

(A43)

where φ_sed_ is the content of organic carbon in sediment (0.033) [1]; ρ_sed_ is density of sediment (1540 g L^-1^), and K_oc_ is listed in **Table A2**.

The change in degradation rate of PCB28 in soil, water and sediment with the temperature was calculated by

$K_{rs}=K_{s}\exp\left[\frac{\Delta E}{R}\left(\frac{1}{T_{0}}-\frac{1}{T_{e}}\right)\right]$ (A44)

where K_rs_ is the temperature-modified degradation rate constant (h^-1^), K_s_ is the reference degradation rate constant at 298 K, ΔE is the activation energy (**Table A2**), R is the gas law constant at 8.314 J K^-1^mol^-1^, T_0_ is the reference temperature (298 K), and T_e_ is the average temperature (K) over the grid cell.

In the atmosphere, degradation rate constants alter not only with temperature fluctuation, but also OH radical concentration [8]. Hence, the following relationship is adopted in this study.

$K_{ra}=K_{r0}\left[OH^{-}\right]10^{6}\exp\left(\frac{\Delta E}{R}\left(\frac{1}{T_{0}}-\frac{1}{T_{e}}\right)\right)$ (A45)

where K_ra_ is a modified degradation rate constant in the atmosphere, K_r0_ is the reference degradation rate constant associated with the concentration of hydroxyl radicals [OH] in the atmospheric compartment. The values of these parameters have been listed in Table A2.

# A2.2 Transport module

A Lagrangian method was employed to calculate the mass exchange of atmospheric horizontal advection for PCB28 between inter-grid cells in the atmosphere. The transport of chemical is driven by wind direction and speed. The distance and concentration of transport are defined as following:

**11**

(1) The distance of transport

The initial-position of emission source is at *S*(t), which corresponding grid cell is (*X*_1_,*Y*_1_). The final position through an advection time step is *S*’(t+δ*t*), which corresponding grid cell is (*X*_i_,*Y*_j_). The average wind speed between (*X*_1_,*Y*_1_) and (*X*_i_,*Y*_j_) is as wind speed of long-range atmospheric transport. According to the amounts of transport grid along *X* and *Y* direction affirm the transport direction and concentration.

$S'_{t+\delta t}(X_{i},Y_{j})=S'_{t+\delta t}(\frac{u_{1}\delta t}{dx},\frac{v_{1}\delta t}{dy})\text{，}(1\leq\frac{u_{1}\delta t}{dx}\leq260,1\leq\frac{v_{1}\delta t}{dy}\leq228)\text{，}$ (A46)

$\left\{\begin{matrix} X_{n}=\frac{(u_{1}+u_{n})\delta t}{2dx}\text{，}\left(1\leq X_{n}\leq260\right)\text{；} \\ Y_{m}=\frac{(v_{1}+v_{m})\delta t}{2dy}\text{，}\left(1\leq Y_{m}\leq228\right)\text{。} \end{matrix}\right.$ (A47)

where $u_{1}$, $v_{1}$ is the velocity of longitudinal and latitudinal direction at initial-position for chemical, respectively. $u_{n}$, $v_{m}$is the velocity of longitudinal and latitudinal direction at final-position for chemical, respectively. $dx$, $dy$is the length of longitudinal and latitudinal direction for a grid cell, respectively. $X_{n}$, $Y_{m}$ is the transported grid amounts of longitudinal (n) and latitudinal (m) direction for chemical, respectively. $(1\leq\frac{u_{1}\delta t}{dx}\leq260,1\leq\frac{v_{1}\delta t}{dy}\leq228)$ is the constraint condition, which the 260 and 228 represent the grid amounts along longitudinal and latitudinal direction, respectively.

(2) The transmission concentration

In general, the transmission concentration decreases with increase of distance from emission source to final destination for chemical. According to the transported grid amounts to define the concentration change of chemical, in the process of calculation, all conditions need to be considered such as positive and negative of wind direction, equal or not equal of the transported grid, etc. Hence, there is a sample to interpret the condition of transmission concentration between source and final destination, as following:

**12**

**12**

$\left\{\begin{matrix} i=\frac{n}{(m+1)}\times j\text{，}(j=0,m+1)\text{；} \\ C_{i+1,j+1}=\frac{q^{(m+2-j)}}{q\times(1.0-q^{(m+2)})\times(1.0-q)^{-1}}\times C_{0}\text{，（}q>1\text{）} \end{matrix}\right.$ (A48)

where *n*, *m* is the transported grid amounts of longitudinal and latitudinal direction from source to final destination, respectively. *Y*_m_, *X*_n_ are both positive transported grid amount, and the *Y*_m_ is bigger than *X*_n_, the transported grid amount is m+2 that including source and final destination. *C*_0_ is initial concentration at source point (mol·m^-3^). *i*, *j*, and *C*_i+1,j+1_ are the grid point between source and final destination and corresponding concentration (mol·m^-3^), respectively. *q* is the ratio of transport, that used to define the transport capacity for different PCB congeners.

The exchange between two atmosphere layers, which is defined by

$f_{a1n}=f_{a2n}=\frac{f_{a1o}Z_{a1}V_{a1}+f_{a2o}Z_{a2}V_{a2}}{Z_{a1}V_{a1}+Z_{a2}V_{a2}}$ (A49)

where *f*_a_ , *Z*_a_ , and *V*_a_ are fugacity, fugacity capacity and volume, respectively. The subscript 1, 2 are the first atmospheric layer (0-1 000 m) and second atmospheric layer (1 000 - 3 000m), respectively. The subscript *o*, *n* represent before and after exchange.

# A3. Model input data

Model input data include daily meteorological data from 1965 to 2010, soil properties, and surface features in the model domain. The detailed information is present in Tian et al. 2011 [1].

**13**

Organic matter content in soil at each grid is assumed by the relationship between organic matter content and organic carbon content [5]:

$O_{m}=\frac{O_{c}}{0.56}$ (A50)

where O_m_ and O_c_ are organic matter content and organic carbon content in soil, respectively.

The surface types, soil properties, and average temperature are illustrated in fig. A3-8


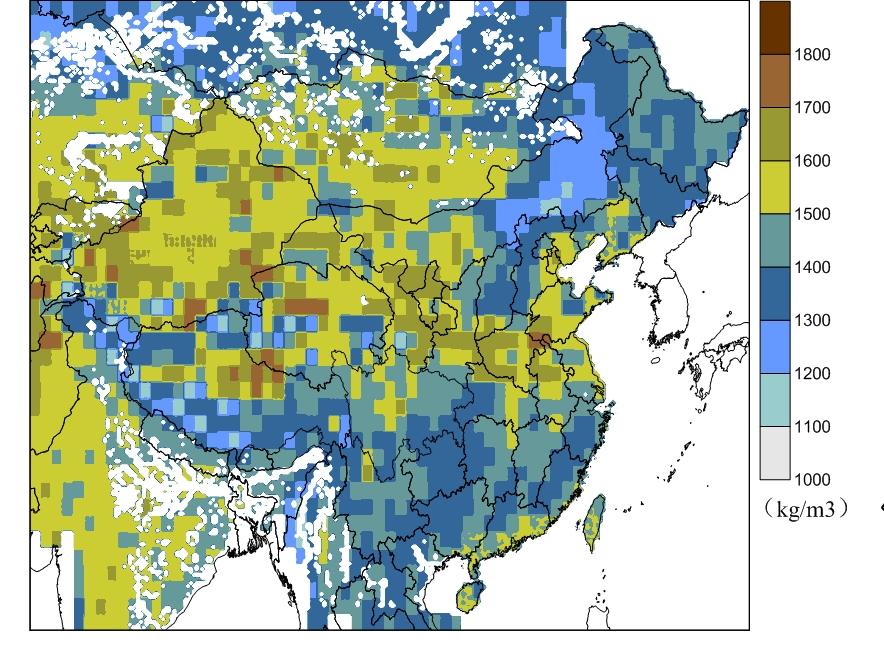


### Figure A3. Gridded soil density in the model domain. The map was drawn by the software of Surfer 9.0, http://www.goldensoftware.com/.


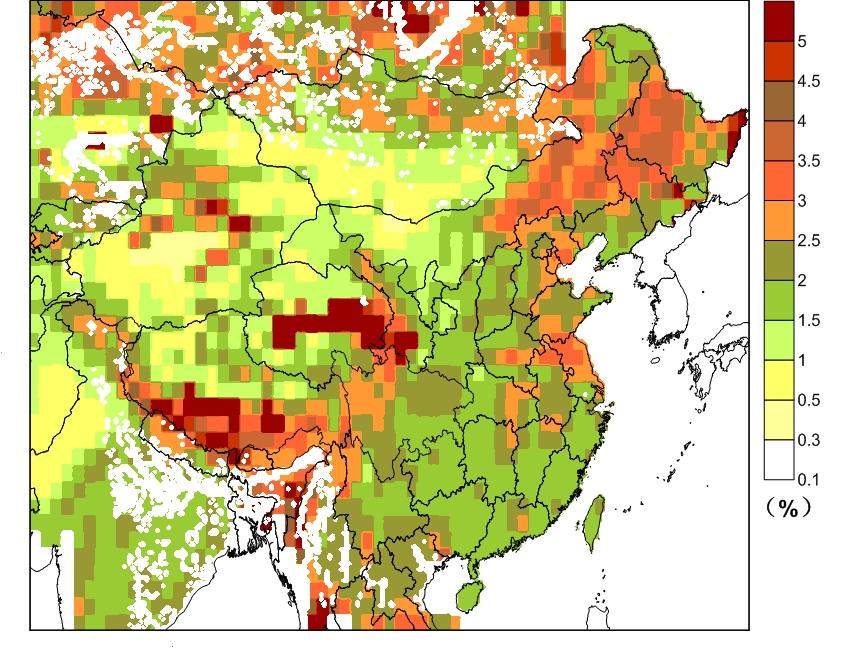


**14**

### Figure A4. Gridded organic carbon content in soil in the model domain. The map was drawn by the software of Surfer 9.0, http://www.goldensoftware.com/.

###
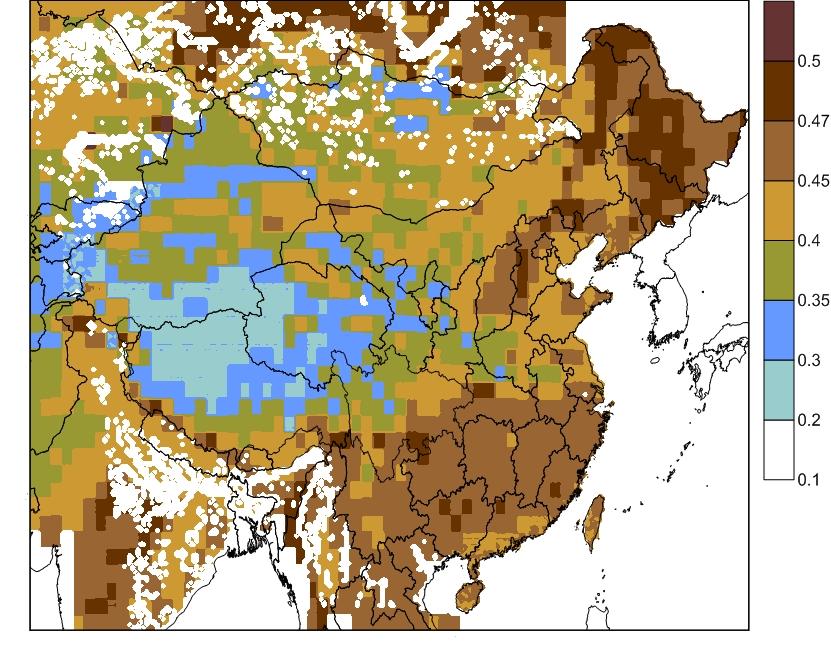


### Figure A5. Gridded soil porosity in the model domain. The map was drawn by the software of Surfer 9.0, http://www.goldensoftware.com/.


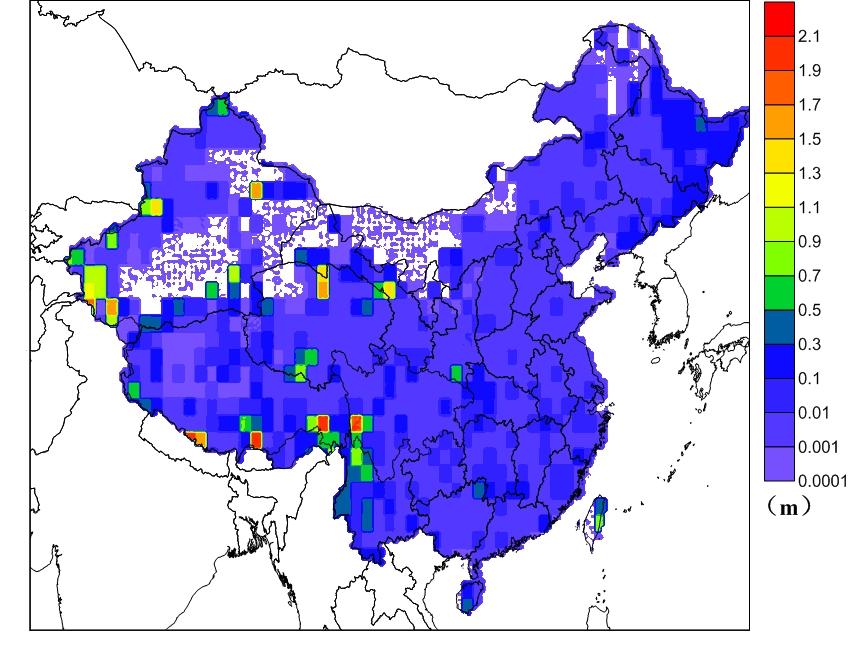


### Figure A6. Gridded surface roughness in the model domain. The map was drawn by the software of Surfer 9.0, http://www.goldensoftware.com/.

**15**


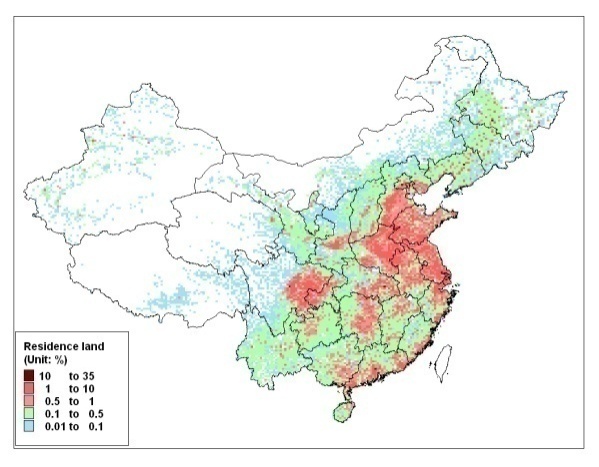
**
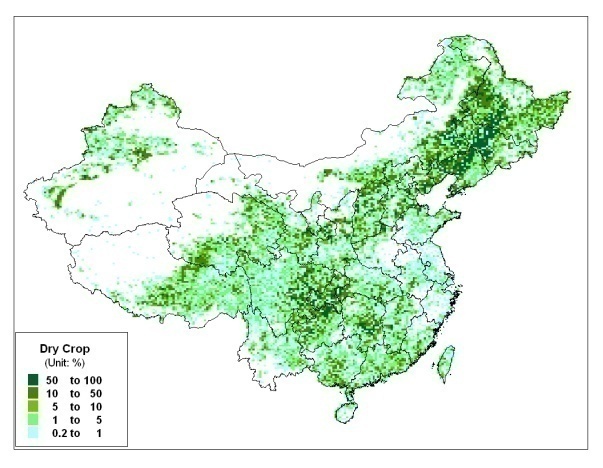
**

(a) (b)

**
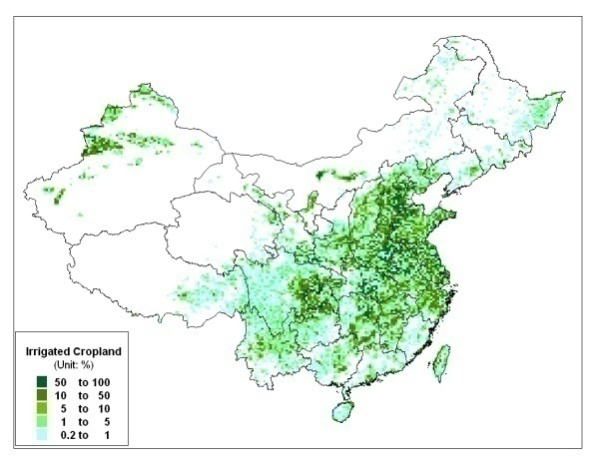

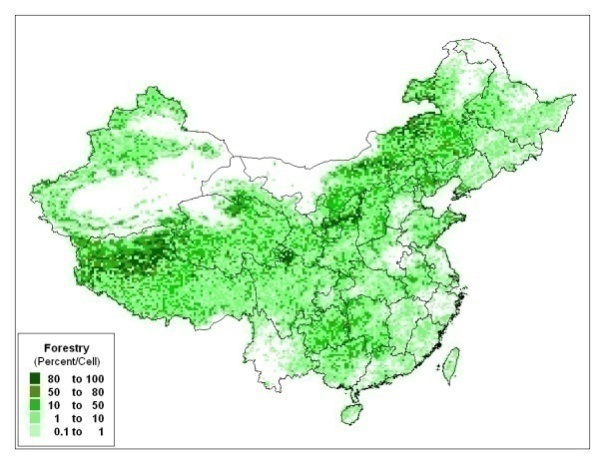
**

(c) (d)

**
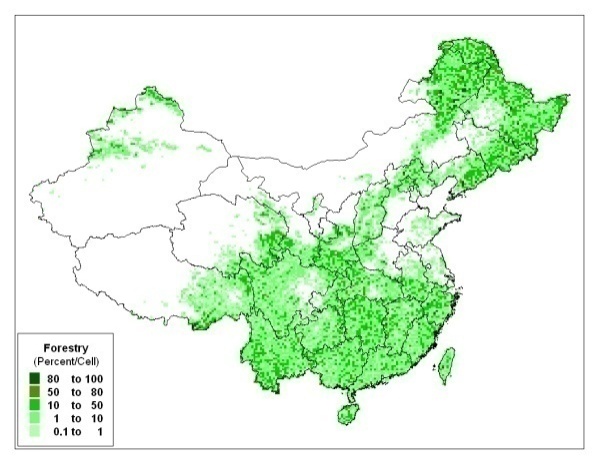
**
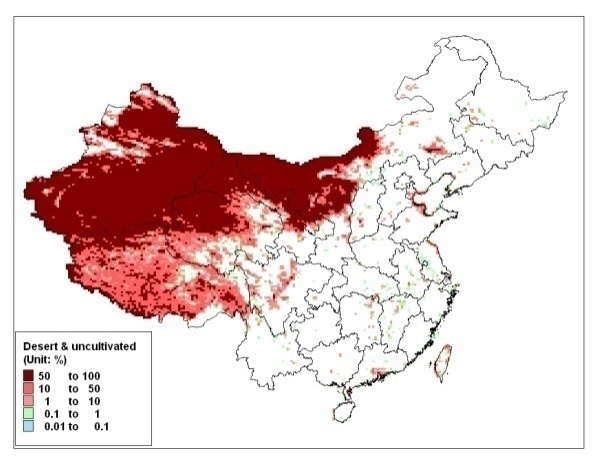


(e) (f)


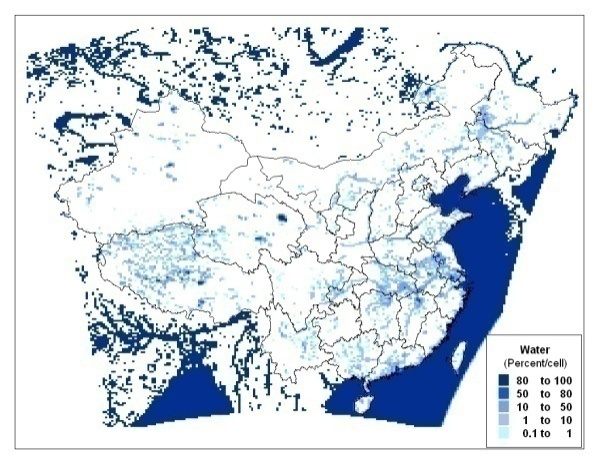


**16**

(g)

### Figure A7. Surface types: urban soil, dry cropland, paddy field, grassland, forest, uncultivated land and water. The maps were drawn by the software of Surfer 9.0, http://www.goldensoftware.com/.

The same soil density, organic carbon content, and soil porosity are applied in all four soil layers. The ratios of volume fractions for air and water are 0.6:0.4 for dry cropland, 0.5:0.5 for paddy field, 0.8:0.2 for urban soil, and uncultured soil, 0.7:0.3 for forestry, and grassland.


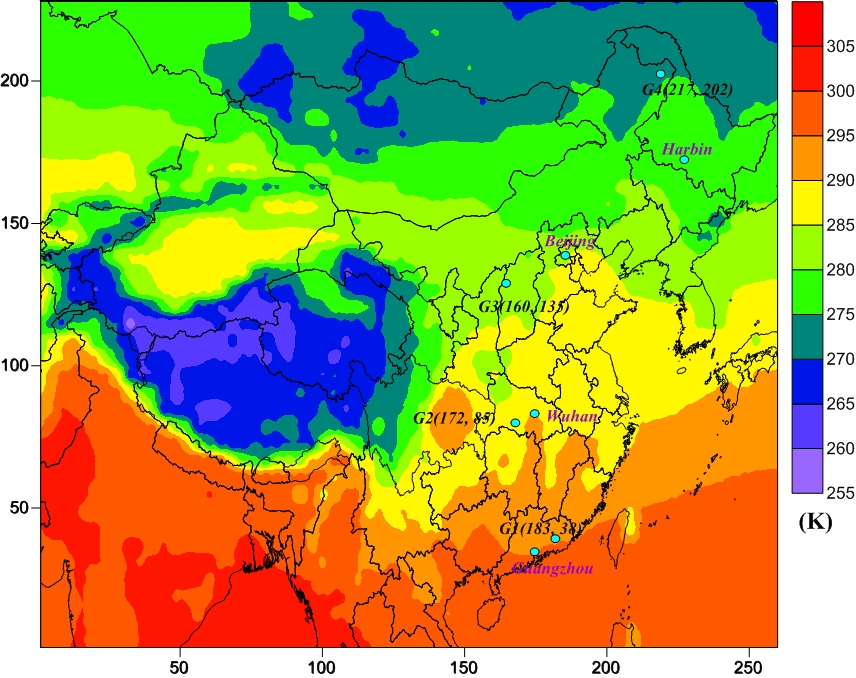


### Figure A8. The annual average temperature in 2005, city sides and grid sides in non-source areas. The map was drawn by the software of Surfer 9.0, http://www.goldensoftware.com/.

# A4. Model evaluation

# A4.1 Comparison between modeled and measured soil concentrations

The monitoring data are from International Joint Research Center for Persistent Toxic Pollutants (IJRC-PTS), the modeled mean concentrations in soil (from top layer to third layer) with 6 soil types which the monitoring sampled soil type is corresponding modeled soil type for PCB28. Calculating Pearson correlations between the measured and modeled soil and air concentrations across China, except individual data that the modeled values are relatively low for rural and background, in which the results of comparison are *R*=0.49, *P*=0.001 for soil.

**17**

Table A3 comparison of modeled and measured concentration in soil (ng/g)

| Name | CH64 | Soil types | Sampling region | Longitude | Latitude | Measure | Model |
| --- | --- | --- | --- | --- | --- | --- | --- |
| DMU29 | 11229154 | Dry cropland | Rural | 110.8888 | 21.67072 | 21.6 | 23.1 |
| DMU33 | 11529951 | Grass | Urban | 118.1611 | 24.74245 | 30.3 | 12.9 |
| CUT19 | 11628132 | Dry cropland | Rural | 100.4861 | 25.35222 | 18.8 | 7.1 |
| CUT18 | 11628313 | Dry cropland | Urban | 102.7046 | 25.04384 | 55.8 | 84.5 |
| CUT11 | 11828362 | Dry cropland | Rural | 102.2653 | 27.89725 | 21 | 10.2 |
| DMU28 | 11830121 | Dry cropland | Urban | 120.2124 | 27.32709 | 35.2 | 15.1 |
| DMU08 | 11929323 | Dry cropland | Rural | 112.5497 | 28.26105 | 13.2 | 26.0 |
| DMU32 | 12128952 | Grass | Urban | 108.4192 | 30.80677 | 37.7 | 20.1 |
| DMU18  DMU27 | 12130154  12230212 | Paddy field  Grass | Rural  Urban | 120.9205  121.3994 | 30.83141  31.11397 | 26.3  29 | 28.5  47.0 |
| DMU14 | 12329563 | Paddy field | Rural | 114.628 | 32.95991 | 17.1 | 17.7 |
| DMU10  CUT22 | 12430131  12528844 | Dry cropland  Dry cropland | Rural  Rural | 120.0552  107.819 | 33.33353  34.50556 | 27.3  10.8 | 47.0  3.9 |
| DMU04 | 12529251 | Dry cropland | Rural | 111.1953 | 34.78077 | 29.4 | 16.9 |
| DMU02 | 12529724 | Paddy field | Rural | 116.7979 | 34.257 | 28.7 | 12.2 |
| DMU30 | 12629734 | Dry cropland | Rural | 116.7737 | 35.44352 | 25.8 | 19.4 |
| DMU25 | 12630032 | Grass | Urban | 119.4124 | 35.46656 | 16.2 | 22.9 |
| CUT21 | 12728124 | Dry cropland | Background | 100.7718 | 36.17359 | 8.6 | 0.6 |
| CUT20  HIT08  HIT18 | 12728244  12728442  12728842 | Dry cropland  Dry cropland  Dry cropland | Rural  Rural  Rural | 101.7875  103.3493  107.3054 | 36.60944  36.59376  36.57517 | 14.1  19.2  15 | 18.8  4.5  2.9 |
| DMU20 | 12730362 | Dry cropland | Rural | 122.305 | 36.87193 | 22.3 | 5.0 |
| DMU09 | 12829332 | Dry cropland | Rural | 112.3928 | 37.48521 | 22.3 | 10.7 |
| DMU01 | 12830024 | Dry cropland | Rural | 119.9773 | 37.32783 | 18.8 | 35.3 |
| DMU13 | 12929742 | Dry cropland | Rural | 116.3819 | 38.53449 | 32.4 | 6.6 |
| HIT17 | 13029054 | Dry cropland | Rural | 109.875 | 39.76508 | 44.9 | 7.9 |
| HIT10 | 13128861 | Dry cropland | Rural | 107.0957 | 40.90446 | 11.1 | 2.1 |
| DMU06 | 13129914 | Dry cropland | Rural | 118.7552 | 40.03898 | 13.1 | 4.4 |
| HIT24 | 13130353 | Paddy field | Rural | 122.7425 | 40.80195 | 9.7 | 4.5 |
| HIT22 | 13230643 | Dry cropland | Rural | 125.5216 | 41.5937 | 6.6 | 2.7 |
| HIT11 | 13329914 | Dry cropland | Rural | 118.8529 | 42.11173 | 18.1 | 1.2 |
| HIT07 | 13330163 | Dry cropland | Rural | 120.6686 | 42.99015 | 21.3 | 4.5 |
| HIT26 | 13526534 | Dry cropland | Rural | 84.894 | 44.43006 | 23.3 | 37.4 |
| HIT03  **18** | 13730831 | Dry cropland | Rural | 127.1215 | 46.43153 | 37 | 6.2 |
| Continuing tableA3 | | | | | | | |
| Name | CH64 | Soil types | Sampling region | Longitude | Latitude | Measure | Model |
| HIT09 | 13830333 | Dry cropland | Rural | 122.7402 | 47.49531 | 27.3 | 0.2 |
| HIT27 | 13831133 | Dry cropland | Rural | 130.5473 | 47.48023 | 23.6 | 0.7 |
| HIT06 | 13831224 | Dry cropland | Rural | 131.934 | 47.24204 | 17.3 | 7.7 |
| HIT20 | 14030621 | Dry cropland | Rural | 125.2139 | 49.17323 | 17.2 | 20.9 |
| HIT37 | 14130531 | Dry cropland | Rural | 124.1397 | 50.42741 | 15.2 | 0.2 |
| HIT12 | 14130541 | forestry | Background | 124.2 | 50.50001 | 27 | 0.1 |

# A4.2 Comparison between modeled and measured air concentrations

The modeled air concentration compared with the measured air concentration from Jaward, et al., [13] for PCB28 in 2004. A comparison is made by calculating Pearson correlations between the modeled and measured air concentrations at the 30 sites (12 rural and 18 urban, except HongKong) with the corresponding grid cells where the sampling sites were located in the same sampling time, in which the results of comparison are for all sites (*R*=0.55, *P*=0.002), for rural sites (*R*=0.66, *P*=0.021), and for urban sites (except Xi’an site because the modeled air concentration is relatively low) (*R*=0.78, *P*=0.000).

The monitoring data are from Hogarh et al., 2012[14], The rural sites were neglected due to they have inadequate data. There are 14 urban sites (except HongKong) with datasets for both air concentrations of trichlorodiphenyl and modeled PCB28 being available. The modeled air concentrations across China are significantly correlated with monitoring data, in which the result of comparison is *R*=0.70, *P*=0.005 for urban sites. The results show that the modeled data agree well with the monitoring data across Chinese air and soil.

**19**

# A5. Air-soil exchange and secondary emissions and residues

# A5.1 Air-soil exchange

The fugacity approach was used to investigate air-soil exchange^5^. We can obtain the soil-air exchange coefficient (*K*_SA_) by using fugacity methods that involve presets conditions for the existence of PCB congeners in air and soils. *K*_SA_ describes the equilibrium portioning of a chemical between air and soil, which is the ratio of the Z values between the soil and air^15^

| **K**_SA_ **= Z**_S_ */* **Z**_A_*= (*φ′_SOM_**K**_OA_/**RT)** / *(*1/**RT***)* ≈ *φ′*_SOM_**K**_OA_ | (A51) |
| --- | --- |

where Z_S_ and Z_A_ represent the fugacity capacity (Z-value, mol·m^-3^·Pa^-1^) in soil and air, respectively; *R* is the gas constant; and *T* is the temperature (K). *φ′*_SOM_ is the mass fraction of SOM (soil organic matter; the soil organic carbon (SOC) content can be estimated as 0.56 of the SOM^5^), and *K*_OA_ is the octanol/air partition coefficient, which is strongly temperature-dependent and spans several orders of magnitude for the range of environmentally relevant temperatures^16,17^.

**Fig. A9** depicts the variation of Log *K*_SA_ with temperature at different SOM contents for PCB28. There is a positive correlation between *K*_SA_ and *K*_OA_. Given a negative correlation between *K*_OA_ and temperature^16^; Log *K*_SA_ increases with decreasing temperature. As shown from **Fig. A9**, *K*_SA_ varies by approximately 5 orders of magnitude, which corresponds to a temperature range of -50 °C to 50 °C. In contrast, there is a positive correlation between *K*_SA_ and SOM because the SOM content affects the accumulation of chemicals. An increase in the SOM content from 10% to 30% will cause a positive change in *K*_SA_ of approximately a half order of magnitude.

**20**

### Figure A9. Variation of the soil-air partition coefficient (KSA) with temperature at different contents of soil organic matter (SOM) for PCB28.

In general, the SOM content and temperature will affect the air-soil exchange of chemicals. In this study, the fugacity fraction (*ff*) was adopted to assess the equilibrium status of a chemical between two interacting phases, in this case soil and air:

| ff = f_S_/(f_S_ + f_A_) (A52) |
| --- |

where f_S_ is the fugacity in the first soil layer (depth of 0.1 cm) in the ChnGIPERM because this layer directly exchanges with the first air layer and f_A_ is the fugacity in the first air layer. Values of ff =0.5 indicate soil-air equilibrium and no net gas exchange. Values > 0.5 indicate net volatilization from the soil, and values < 0.5 indicate net deposition to the soil (in a way, this phenomenon also represents primary emissions due to direct emissions to air from PCB-containing products).

# A5.2 Secondary emissions and residues

The soil retention capacity (SRC) and temperature govern how much of a chemical is able to accumulate in soil during the primary emission and deposition period and how much were released back to the atmosphere, a phenomenon called secondary emission that results in deposition elsewhere through long- or short-range atmospheric transport of chemicals. The *EF* (emission factor, the ratio between secondary emissions and residues) of PCB28 at these 4 sites (in non-source areas) are depicted in Fig. A10.

**21**

### Figure A10. Monthly variation of the secondary emission factors (*EF*) in G1 (183, 38), G2 (172, 85), G3 (160, 135) and G4 (217, 202) in non-source areas.

**References**

1. Tian, C., Liu, L., Ma, J., Tang, J. & Li, Y. Modeling redistribution of α-HCH in Chinese soil induced by environment factors. *Environ. Pollut*. 159, 2961-2967 (2011).
2. Xu, Y. et al. Assessing Environmental Fate of β-HCH in Asian Soil and Association with Environmental Factors. *Environ. Sci. Technol*. 46, 9525-9532 (2012).
3. Tian, C., Li, Y.F., Wu, H. & Ma, J. Modelling historical budget of α-hexachlorocyclohexane in Taihu Lake, China. *Chemosphere*, 77, 459-464 (2009).
4. Harner, T., Bidleman, T., Jantunen, L. & Mackay D. Soil-air exchange model of persistent pesticides in the United States cotton belt. *Environ. Toxicol. Chem.* 7, 1612-1621 (2001).
5. Mackay D. Multimedia Environmental Models: the Fugacity Approach, CRC Press, Boca Raton, FL, (2001).
6. Mackay, D., Shiu, W.Y., Ma, K.C. & Lee, S.C. Handbook of physical-chemical properties and environmental fate for organic chemicals (CRC) 2nd Ed, (2006).

**22**

1. Wania, F., Mackay, D., McLachlan, M.S., Sweetman, A. & Jones, K.C. Global modelling of polychlorinated biphenyls. WECC report, 1- 22(1999).
2. Gong, S.L. et al. GEM/POPs: a global 3-D dynamic model for semi-volatile persistent organic pollutants - Part 1: Model description and evaluations of air concentrations. *Atmos. Chem. Phys*. 7, 4001-4013 (2007).
3. Beyer, A., Wania, F., Gouin, T., Mackay, D. & Matthies, M. Selecting internally consistent physicocemical properties of organic compounds. *Environ. Toxicol. Chem.* 21, 941-953(2002).
4. Harner, T. & Bidelman, T.F. Measurements of octanol-air partition coefficients for polychlorinated biphenyls. *J. Chem. Eng. Data* 41, 895-899(1996).
5. Schwarzenbach, R.P., Gschwend, D.M. & Imboden, D.M. Environmental Organic Chemistry Wiley Interscience, New York, (1993).
6. Wu S. & Gschwend, P. Numerical modeling of sorption kinetics of organic compounds to soil and sediment particles. *Water Resour. Res.* 24, 1373-1383(1988).
7. Jaward, F.M. et al. Passive air sampling of polychlorinated biphenyls, organochlorine compounds, and polybrominated diphenyl ethers across Asia. *Environ. Sci. Technol.* 39, 8638-8645 (2005).
8. Hogarh, J.N. et al. Passive air monitoring of PCBs and PCNs across East Asia: A comprehensive congener evaluation for source characterization. *Chemosphere*, 86, 718-726 (2012).
9. Li, Y.F. et al. Polychlorinated biphenyls in Global Air and surface Soil: Distributions, Air-Soil exchange, and fractionation effect. Environ. Sci. Technol. 44, 2784-2790 (2010).
10. Harner, T. & Bidleman, T.F. Measurements of octanol-Air partition coefficients for polychlorinated biphenyls. J. Chem. Eng. Data 41, 895-899 (1996).
11. Shoeib, M. & Harner, T. Characterization and comparison of three passive air samplers for persistent organic pollutants. Environ. Sci. Technol. 36, 4142-4151 (2002).

**24**

**23**

**23**
